# Changes and Appointments

**Published:** 1850-01

**Authors:** 


### THE MEDICAL EXAMINER.

PHILADELPHIA, JANUARY, 1850.

We present the greetings of the season to our readers with the opening number of a new volume. When we assumed the Editorial charge of this Journal, we did so with a diffidence and conviction of the responsibilities of a journalist that the experience of the by-gone year has not diminished. We then assured our readers, and those whose works might be presented to us for criticism, that the course of the Examiner would be impartial and independent; and that whilst we would "nothing extenuate," neither would we "aught set down in malice." This intention we have honestly endeavored to fulfil.

We will endeavor to pursue the same course during the present year. At the same time it is our earnest wish to avoid all useless discussions and controversies, especially such as are of a personal nature, believing that they never result in anything but harm, both to the participators and the cause. Our pages will always be open, however, to the temperate consideration of all matters appertaining to medical science.

Much of the original matter published during the past year has been extensively copied into the domestic and foreign journals,—a gratifying evidence of its merits and usefulness. There is still much more in the unpublished experience of our city and country practitioners that needs but to be brought to light to establish its value.

Whilst we are truly grateful to the profession for the kind support that we have received from them, we solicit a continuance of the same in the form of contributions to our pages; assuring them, in return, that no efforts shall be wanting on our part to render the Examiner both useful and acceptable to them.

### CHANGES AND APPOINTMENTS.

Dr. Samuel George Morton was unanimously elected President of the Academy of Natural Sciences of Philadelphia, at the election December 25th, ult.

Prof. Shotwell has been transferred to the chair of Materia Medica in the Medical College of Ohio, vacated by the death of Prof. Harrison.

Dr. Wm. V. Keating has been appointed Lecturer on Obstetrics in the Philadelphia Association for Medical Instruction, to fill the vacancy caused by the appointment of Prof. Tucker to the chair of Practice in Hampden Sidney College.

Dr. Paul F. Eve has retired from the Southern Medical and Surgical Journal, in consequence of ill health and other afflictions. We sincerely sympathise with Dr. E., and trust that brighter days are yet in store for him. Dr. I. P. Garvin succeeds him.

There are three Medical Colleges now in operation in Indiana—one located at each extreme, and one in the centre of the State.

St. Vincent's Hospital is the title of a new hospital recently organized in New York City, under the charge of the Sisters of Charity. The medical staff consists of Wm. Power, M. D. and Wm. Murray, M. D., Attending Physicians. Richard Fraser, M. D., Visiting Physician. Wm. H. Van Buren, M. D., J. W. Schmidt, Jr., M. D., Attending Surgeons. Valentine Mott, M. D., Consulting Surgeon.

Drs. Gaillard and De Saussure have retired from the Editorship of the Charleston Medical Journal. This has ever been one of our favorite exchanges, and we have always regarded its Review department among the very best in the country; we part with them, therefore, as valued friends, whose place it will be difficult to supply.

